# Effects of qigong exercise on the physical and mental health of college students: a systematic review and Meta-analysis

**DOI:** 10.1186/s12906-022-03760-5

**Published:** 2022-11-08

**Authors:** Jianping Lin, Yi fang Gao, Yue Guo, Ming Li, Yuxiang Zhu, Ruoshi You, Shaoqing Chen, Shizhong Wang

**Affiliations:** 1 School of Health, Fujian Medical University, Fuzhou, China; 2 College of Rehabilitation Medicine, Fujian University of Traditional Chinese Medicine, Fuzhou, China; 3 Department of Rehabilitation,The First Affiliated Hospital of Fujian Medical University, Fuzhou, China; 4 Fujian Provincial Collaborative Innovation Center of Geriatric Rehabilitation and Industry Promotion, Fuzhou, China

**Keywords:** Qigong exercise, College students, Physical fitness, Mental health

## Abstract

**Background:**

Physical and mental health problems are becoming more serious among college students due to lifestyle changes and increased academic stress. Qigong exercise has been regarded as a potentially effective intervention to improve the physical and mental health of college students.

**Methods:**

Eleven databases were searched from their respective inception dates to April 2022. Relevant randomized controlled trials (RCTs) were included. Physical and psychological conditions, including limb muscle strength, flexibility, cardiorespiratory endurance, vital capacity, blood pressure and heart rate, as well as depression, anxiety and mood, were evaluated. The risk of bias was assessed with the Cochrane Collaboration tool.

**Results:**

Sixteen randomized controlled trials were included in the meta-analysis. Significant improvements in cardiorespiratory endurance (MD = 3.83, 95% CI: 0.99 to 6.67, *P* = 0.008) and flexibility (MD = 3.01, 95% CI: 1.21 to 4.81, *P* = 0.001) were observed. We also observed that Qigong exercise significantly reduced depression and anxiety symptoms (SMD=-0.89, 95% CI: -1.17 to -0.61, *P* < 0.00001; SMD=-0.78, 95% CI: -1.31 to -0.25, *P* = 0.004). Nevertheless, no significant effects on muscle strength, vital capacity, blood pressure, heart rate or mood were found.

**Conclusion:**

Qigong exercise was advantageous for college students in terms of improving flexibility and cardiorespiratory endurance and alleviating depression and anxiety to some extent. However, due to the limited number of eligible trials and the low methodological quality, more well-designed RCTs are needed in the future.

**Supplementary Information:**

The online version contains supplementary material available at 10.1186/s12906-022-03760-5.

## Introduction

With the number of cellphone users increasing dramatically [1], an increasing number of young people are spending a lot of time on electronic devices, resulting in a sedentary lifestyle being prevalent among college students worldwide. A report among East Asian college students indicated that physical inactivity is present in 7.2% in Singapore, 16.8% in Hong Kong, and 28.5% in South Korea [2]. In 2018, a study of 1.9 million participants from 168 countries revealed that more than a quarter of adults worldwide fail to achieve the recommended level of exercise [3]. Similarly, in the United States, fewer than 60% of students perform 30 min of moderate-intensity exercise a day [4]. Sedentary lifestyle is increasingly prevalent and has also been recognized as an important risk factor for chronic conditions such as cardiovascular disease, metabolic syndrome and diabetes [5–8].

Meanwhile, studies have shown that college students may have ‘worse’ mental health than the general population [9]. Compared with nonstudent groups of the same age, college students are more likely to suffer from a variety of mental health problems due to the influence of multiple pressures, such as academic challenges and competition with peers [10–12]. A survey of 14,175 college students in the United States revealed that the morbidity of depression was 17.3%, that of anxiety was 7.0%, that of suicidal ideation was 6.3%, and that of nonsuicidal self-harm was 15.3% [13]. In a 2017 global study, 35% of 13,984 college students surveyed worldwide were positive for at least one of the mental disorders when assessed [14]. In addition, the proportion of college students experiencing depressive and anxiety symptoms has been increasing over the past decade [15], and these psychological problems are associated with poorer academic performance, unstable intimate relationships, and even suicidal behaviour [16]. If mental health problems are ignored and untreated, they may lead to students suffering from dropping out of college, committing suicide, or engaging in other risky behaviours [17]. Due to the negative impact on individuals and society, the mental health of college students has received much attention from society in the past 20 years and has become a hot topic in the field of psychology.

Qigong is a traditional Chinese aerobic exercise for physical and mental health, based on Taoist philosophy and traditional Chinese medicine theory [18]. It is a total body and mind movement that coordinates body posture, movement and breathing and has been used for thousands of years in China to promote health [19–20]. As a mind-body exercise, the key elements of Qigong are body movement, spiritual guidance and controlled breathing [21–22], and there are various forms of Qigong in China, including Tai Chi [23],Wuqinxi, Baduanjin, Yijinjing, and Liuzijue [24]. Previous studies have indicated that Qigong can enhance the cardiorespiratory endurance and flexibility of college students, reduce anxiety, alter their state of mind, and improve the psychological well-being, thus promoting the development of their physical and mental health [4, 9, 25–26]. However, no previous review systematically assessed Qigong exercise for physical and mental health among college students. Although a narrative review analysed Tai Chi and Qigong for the treatment and prevention of mental disorders, it lacked a quantitative analysis [27]. A meta-analysis assessed the effect of Qigong exercise on psychological status in adolescents, but it was not based on RCTs and did not contain an analysis of physical functions [28]. Meanwhile, there are some studies containing meta-analyses of RCTs that have evaluated the effects of Qigong on physical [29–30] and mental health [31–34], but they focused mainly on middle-aged and elderly people with various diseases, with only a small part of them being young people. Therefore, this study aimed to conduct a comprehensive systematic review and meta-analysis to evaluate the effects of Qigong exercise on the physical and mental health of college students.

## Methods

### Search strategy

Original research articles published from database inception to April 2022 were identified with keywords such as “Qi gong”, “mindful exercise”, “college students” and “random” from the following 11 electronic databases: PubMed, Cochrane Central Register of Controlled Trials (CENTRAL), Embase, Web of Science, Ovid, ClinicalTrials.gov, CINAHL (via EBSCOhost), EBSCO, China National Knowledge Information Database (CNKl), Chinese Scientific Journal Database (VIP) and WanFang Database. The journal languages were restricted to Chinese and English. The inclusion and exclusion criteria were as follows: (1) RCTs published in English and Chinese; (2) Qigong (including Tai Chi, Wuqinxi, Baduanjin, Yijinjing, and Liuzijue) as an intervention to improve physical and mental health; (3) Outcomes included commonly psychological indicators (depression, anxiety and mood) or physical fitness (limb muscle strength, flexibility, cardiorespiratory endurance, vital capacity, blood pressure and heart rate); and (4) participants were college students irrespective of health status. Those studies not focusing on Qigong or Qigong mixed with other interventions, with no full text and with insufficient information and data were excluded from our analysis. The Preferred Reporting Items for Systematic Reviews and Meta-Analyses (PRISMA) guidelines were used to present detailed results. The PROSPERO registration number for this systematic review is CRD42021256823.

### Study selection

Two reviewers independently screened all study titles and abstracts. The full text of the studies that potentially met the inclusion criteria was obtained, and all potentially relevant references were retrieved according to the predefined inclusion criteria. Disagreements were resolved through discussion and, if necessary, consultation with a third investigator to reach a consensus.

### Data extraction

Two reviewers independently selected studies based on the inclusion and exclusion criteria. The following details of each study were extracted: (1) general information: study title, first author name and year of publication; (2) characteristics of participants: mean age, sample size; (3) characteristics of included studies: study type and design, interventions in experimental and control groups, duration of treatment; and (4) outcome measures and results: mean, SD (standard deviation). If data were missing from the included studies or the results were not reported as the mean and standard deviation, the authors were contacted by email for data.

### Methodological Quality Assessment

The quality of each study was assessed independently by 2 reviewers according to the Cochrane Collaboration tool [35]; when the opinions of the 2 reviewers were inconsistent, a third reviewer was consulted, and a consensus was reached through discussion.

### Statistics

Cochrane Collaboration Review Manager Software (RevMan version 5.3.0) was used for all data analyses. For continuous data, when the units or methods for evaluating the same outcome were consistent, the mean difference (MD) was used as the summary statistic; otherwise, standardized mean differences (SMD) were used when the units or methods for evaluating the same outcome were inconsistent. Heterogeneity was assessed by calculating I^2^ values. According to the study of Borenstein et al. 2010 and Handbook for Systematic Reviews,a random effects model was used to pool the data as taking variability between the included studies into consideration [36, 37]. *P values* < 0.05 were considered to be statistically significant. Funnel plot analysis was performed when at least 10 studies were included in the analysis [38].

## Results

### Study selection

**Fig. 1:**
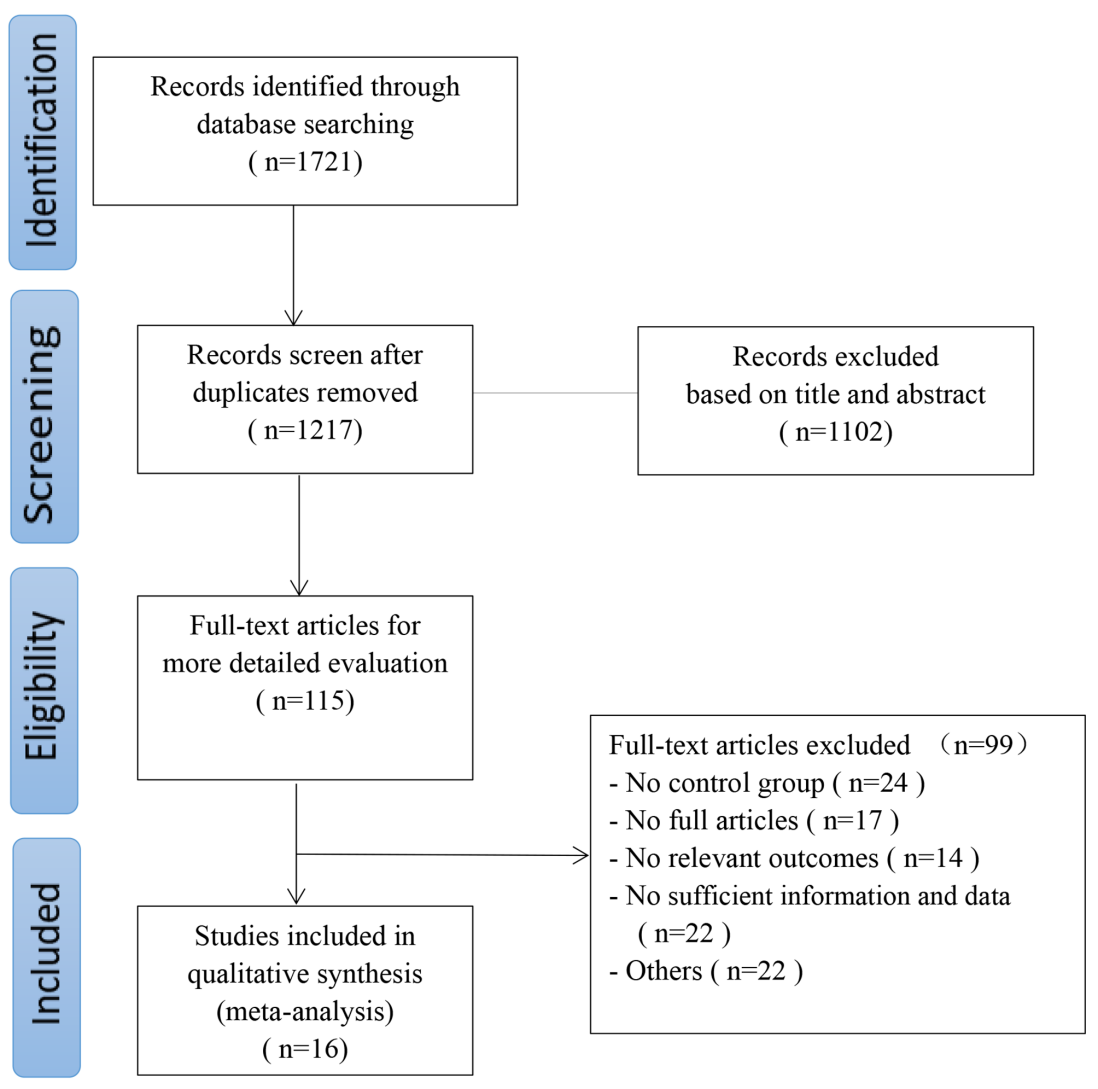
Flowchart of the search process for the articles

A total of 1721 relevant articles were retrieved from eleven electronic databases. After removing duplicate articles, 1217 articles remained. After reading the titles and abstracts, 1102 articles were excluded. After reading the full texts of the remaining 115 articles, 99 studies were further excluded for the following reasons: insufficient information and data (n = 22); no relevant outcomes (n = 14); no control group (n = 24); not a full article (n = 17); and other (n = 22). Finally, 16 studies were found to meet the study inclusion criteria and were pooled for meta-analysis [4, 9, 39−52]. The PRISMA flow diagram is illustrated in Fig. 1.

### Study characteristics

The basic characteristics of the included studies are summarized in Table 1. A total of 1628 subjects in sixteen studies from thirteen Chinese [39–46, 48−52] and three English articles [4, 9, 47] were included in the final analysis. All these studies were conducted in China and published in Chinese and foreign language journals from 2015 to 2022. All participants were undergraduate or graduate students. The studies compared the effects of Qigong exercise with original sports exercise practices, muscle relaxation training or maintaining the original lifestyle without any intervention on the physical and mental health of college students. The intervention time in the Qigong group and the control group was the same, and interventions ranged from 8 weeks to 32 weeks, with most interventions being 12 weeks. All studies reported physical or psychological outcomes. Among them, in terms of psychological indicators, eleven studies evaluated depression by using the self-rating depression scale (SDS) [39, 41, 48, 50], SCL-90 Depression [42–43, 49, 51–52], center for epidemiologic studies depression scale (CES-D) [46] and hamilton depression scale (HAMD) [45]; eight studies used the self-rating anxiety scale (SAS) [39, 48, 50] and SCL-90 anxiety [42–43, 49, 51–52] to assess anxiety, and three studies used the profile of mood states (PMOS) [4, 47, 49] to assess mood. In the evaluation of physical indicators, seven studies reported vital capacity [4, 9, 40, 42–44, 52], five studies used a standing long jump to evaluate lower limb strength [4, 40, 42–43, 52], four studies reported hand grip force levels [4, 42–43, 52], five studies reported using the Sit-and-Reach test to assess flexibility [4, 9, 40, 42–43], five studies reported using the step test to assess cardiorespiratory endurance [4, 9, 42–43, 52], three studies reported blood pressure [4, 9, 44], and three studies reported heart rate [4, 9, 44].

**Table 1:** Characteristics of the included studies

First author, year	Age (years) mean ± SD		Sample size		Intervention		Outcome	Treatment duration
	QG	CG	QG	CG	QG	CG		
Cheng X, [45] 2016	21.1 ± 1.4	21.0 ± 1.6	15	15	3 × (40–60)min/wk for 12 weeks(Wuqinxi)	No intervention	HAMD	12 weeks
Chen J, [46] 2019	NR		18	18	3 × 60 min/wk for 16 weeks(Tai Chi)	No intervention	CES-D	16 weeks
Chen T, [47] 2016	22.5 ± 2.0		21	21	5 × 90 min/wk for 8 weeks(Baduanjin)	Relaxation training	POMS	8weeks
Jiao X, [52] 2021	20.0 ± 1.3		40	40	5 × 80 min/wk for 16weeks(Wuqinxi)	5 × 80 min/wk for 16weeks (Original sports )	Vital capacity, Standing long jump, Hand grip force, Step test, SCL-90Depression, SCL- 90 Anxiety	16weeks
Guo T, [51] 2021	NR		30	30	3 × 45 min/wk for 12weeks(Baduanjin)	No intervention	SCL-90 Depression, SCL-90 Anxiety	12weeks
Ke X, [39] 2019	19.4 ± 0.5	19.5 ± 0.6	20	17	≥ 5 × 60 min/wk for 10 weeks(Baduanjin)	No intervention	SDS, SAS	10 weeks
Li M, [4] 2015	20.63 ± 1.03	20.92 ± 1.15	101	105	5 × 60 min/wk for 12 weeks(Baduanjin)	No intervention	Vital capacity, Standing long jump, Hand grip force, Sit-and-Reach, Step test, Blood pressure, Heart rate, POMS	12weeks
Lai Q, [40] 2018	20.61 ± 1. 06	20.54 ± 1. 05	30	30	5 × 60 min/wk for 12 weeks(Baduanjin)	No intervention	Vital capacity, Standing long jump, Sit-and-Reach	12weeks
Luo S, [50] 2021	NR		157	158	3 × 30 min/wk for 16weeks(Tai Chi)	No intervention	SDS, SAS	16 weeks
Wang M, [41] 2020	NR		30	30	7 × 40 min/wk for 24weeks(Wuqinxi)	No intervention	SDS	24 weeks
Wei Q, [42] 2017	NR		60	60	5d×2times/wk for 12weeks(Yijinjing)	No intervention	Vital capacity, Standing long jump, Hand grip force, Sit-and-Reach, Step test, SCL-90 Depression, SCL-90 Anxiety	12 weeks
Wang B, [49] 2021	NR		100	100	≥ 3 × 90 min/wk for 18weeks(Baduanjin)	No intervention	POMS, SCL-90 Depression, SCL-90 Anxiety	18 weeks
Yan H, [43] 2017	NR		50	50	5d×2times/wk for 12weeks(Baduanjin)	No intervention	Vital capacity, Standing long jump, Hand grip force, Sit-and-Reach, Step test, SCL-90 Depression, SCL-90 Anxiety	12 weeks
Yuan M, [44] 2017	NR		12	12	4 × 60 min/wk for 20weeks(Tai Chi)	No intervention	Vital capacity, Blood pressure,Heart rate	20 weeks
Zheng G, [9] 2015	20.7 ± 1.1	20.6 ± 1.2	95	103	5 × 60 min/wk for 12weeks(Tai Chi)	No intervention	Vital capacity, Sit-and-Reach, Step test, Blood pressure, Heart rate	12 weeks
Zhang Y, [48] 2021	19–22		30	30	2 × 90 min/wk for 8weeks(Baduanjin)	No intervention	SDS, SAS	8weeks

SD:standard deviation;QG: qigong group;CG: control group; NR: not reported; POMS:Profile of mood states;SDS:Self-rating depression scale;SAS:Self-rating anxiety scale;HAMD:Hamilton depression rating scale;CES-D:Center for epidemiologic studies depression scale

### Assessment of risk of bias

**Fig. 2:**
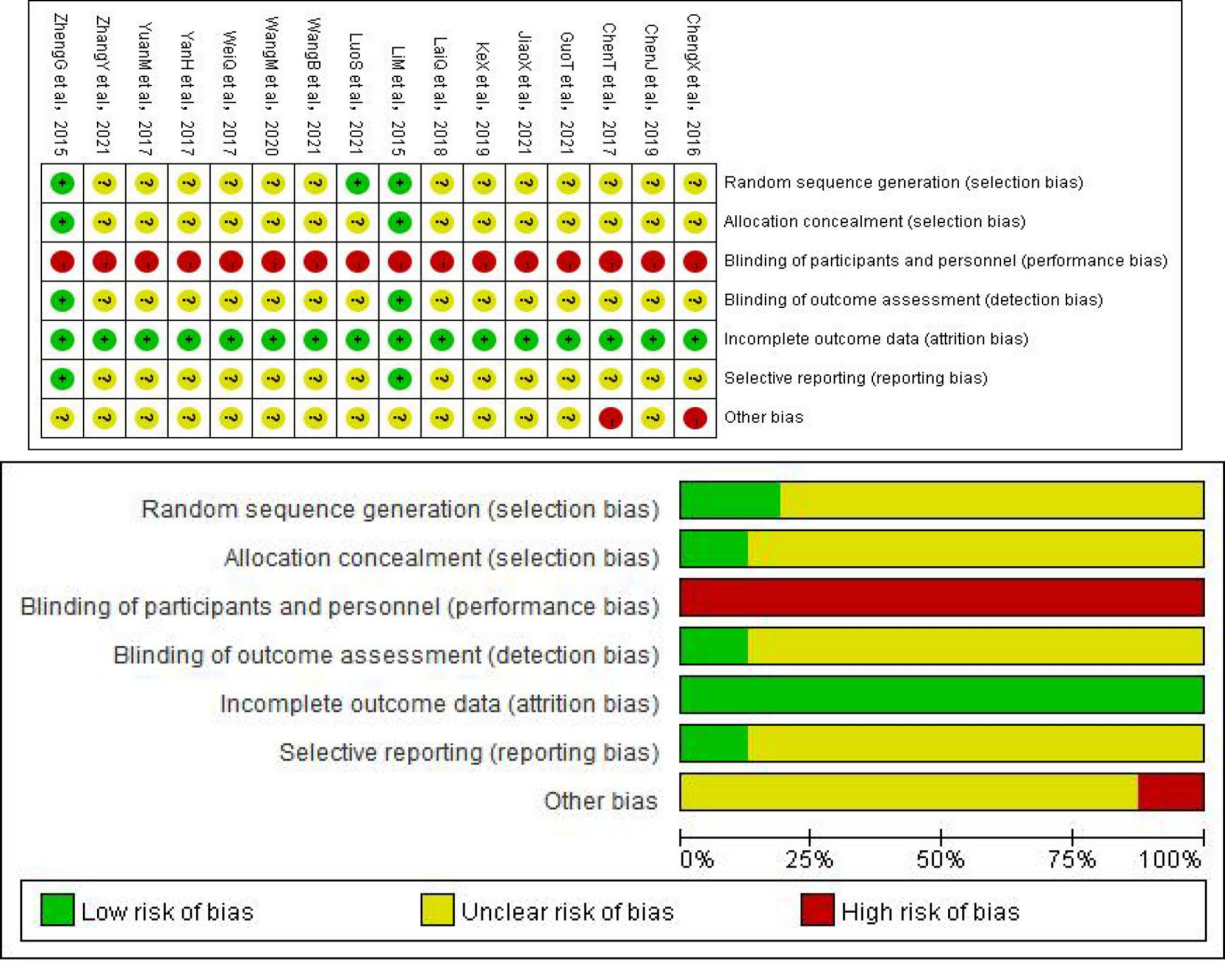
Risk of bias summary and graph

All 16 studies reported randomization, but only 3 trials reported randomizing the patients by using statistical software or a random number table to generate randomization sequences [4, 9, 50]. Two studies mentioned allocation concealment. The allocation sequence was concealed with password access files or kept with a project manager [4, 9]. For participant and personnel blinding, 16 studies were rated as high risk due to completely different intervention methods between the experimental and control groups [47, 52] and no intervention measures in control groups [4, 9, 39–46, 48−51]. Thus, it was unable to do blindness. Two studies reported dropouts during the clinical study, but all indicated reasons were unlikely to be related to the results [4, 9]. Therefore, the risk of bias for incomplete outcome data was assessed as low risk for all studies. Two studies were assessed as low risk with a protocol available [4, 9], while the remaining studies were unclear, as there was not enough information to judge. Two studies described the limitations of the study in the Discussion section and were therefore rated as high risk [45, 47] (Fig. 2).

### Evaluation of physical outcomes

#### Flexibility

**Fig. 3:**
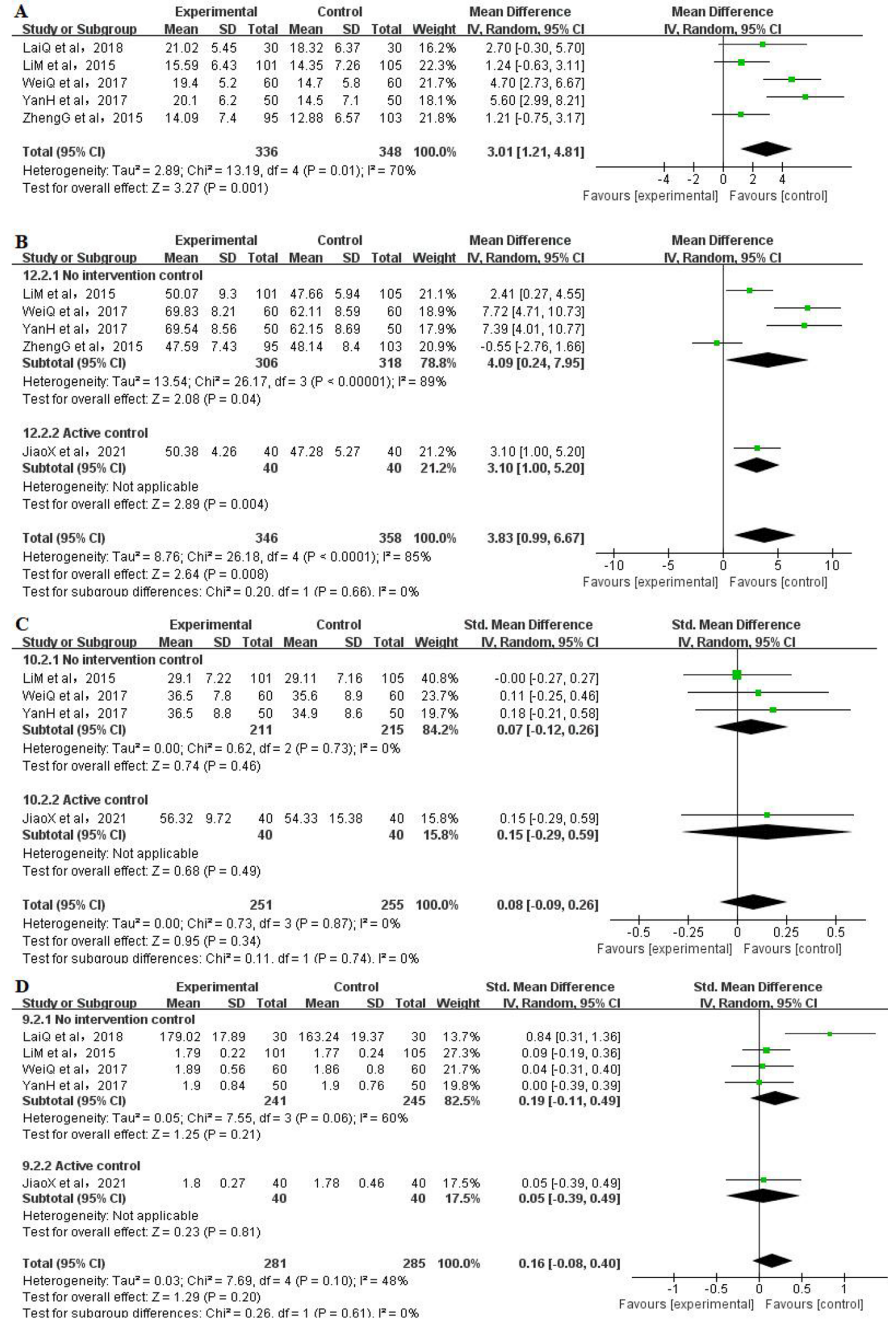
Meta-analysis of Qigong exercise on physical fitness (A:flexibility, B:cardiorespiratory endurance, C:hand grip force, D:standing long jump)

Five RCTs [4, 9, 42–43] analysed the scores of the Sit-and-Reach test as indicators of flexibility, including a total of 684 participants (336 in the Qigong group and 348 in the control group). The pooled results revealed a significant difference for the effect of Qigong exercise on the sit-and-reach index (MD = 3.01, 95% CI: 1.21 to 4.81, *P* = 0.001). However, due to the high heterogeneity among the studies (I^2^ = 70%) (Fig. 3A), a sensitivity analysis was conducted in which the included studies were excluded one by one. However, no matter which study was removed, the heterogeneity remained high. The Qigong group was also better than the control group (*P* < 0.05).

### Cardiorespiratory endurance

The scores of the step test for cardiorespiratory endurance were analysed in five RCTs [4, 9, 42–43, 52] that included a total of 704 participants (346 in the Qigong group and 358 in the control group). Compared to the control group, the results of the pooled meta-analyses showed a significant improvement (MD = 3.83, 95% CI: 0.99 to 6.67, *P* = 0.008) in college students with Qigong exercise. Although the subgroup analysis also showed that Qigong significantly increased cardiorespiratory endurance, the heterogeneity remained high (I^2^ = 89%) (Fig. 3B). And heterogeneity did not obviously change after sensitivity analysis by removing any one of those studies.

### Hand grip force

Four RCTs [4, 42–43, 52] analysed the effect of Qigong interventions on hand grip force levels, including a total of 506 participants (251 in the Qigong group and 255 in the control group). The pooled result showed no statistically significant difference between Qigong and control groups (SMD = 0.08, 95% CI: − 0.09 to 0.26, *P* = 0.34, I^2^ = 0%) (Fig. 3C). And the subgroup meta-analysis showed that there was no significant difference in neither no intervention control subgroup (*P* = 0.46) nor active control subgroup (*P* = 0.49).

### Standing long jump

The standing long jump scores were analysed in five RCTs [4, 40, 42–43, 52] involving a total of 566 participants (281 in the Qigong group and 285 in the control group). The pooled results showed that the difference was not statistically significant (SMD = 0.16, 95% CI: − 0.08 to 0.40, *P =* 0.20, I^2^ = 48%) (Fig. 3D). And the subgroup meta-analysis showed that there was no significant difference in neither no intervention control subgroup (*P* = 0.21) nor active control subgroup (*P* = 0.81).

### Vital capacity

**Fig. 4:**
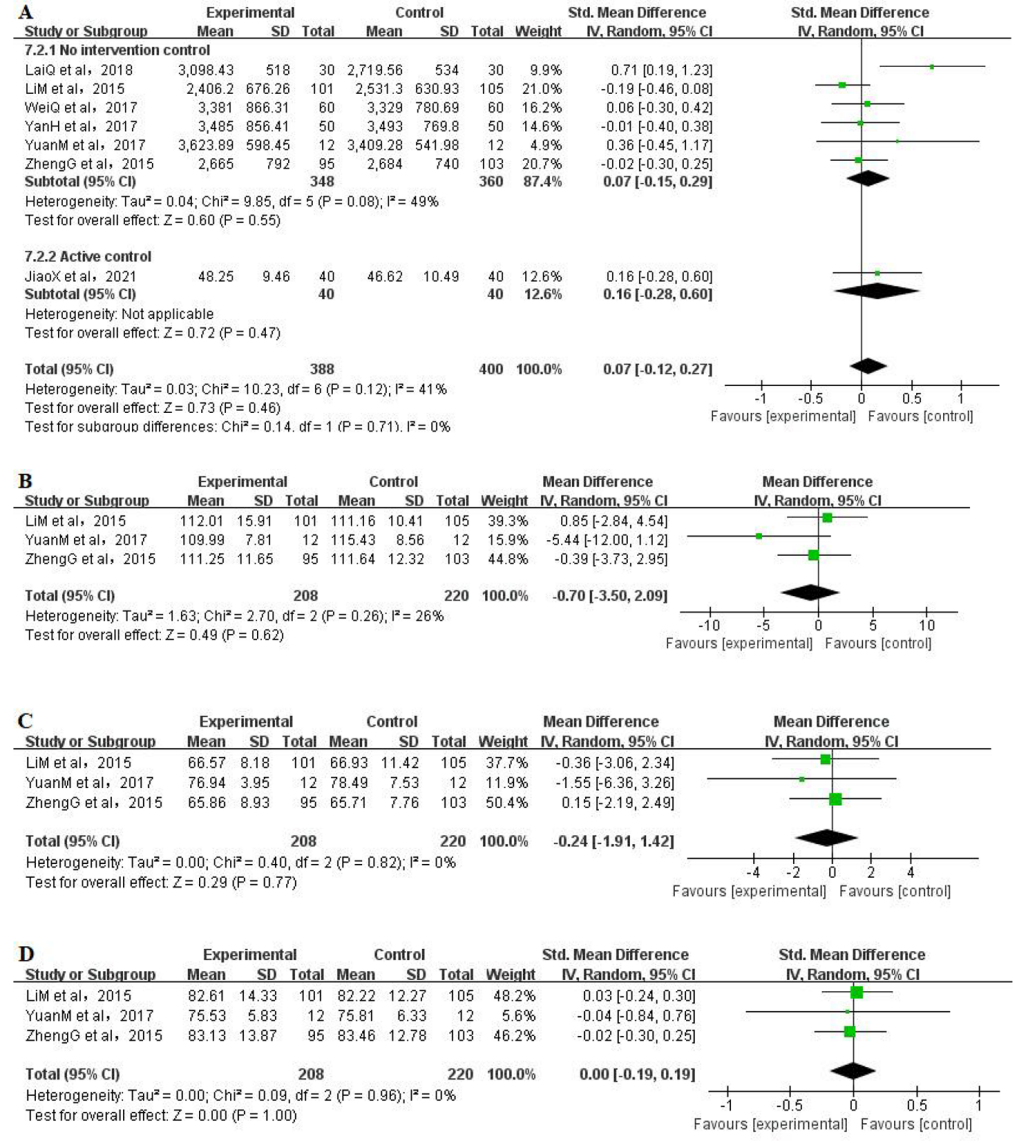
Meta-analysis of Qigong exercise on vital capacity,blood pressure and heart rate (A:vital capacity, B:systolic blood pressure, C:diastolic blood pressure, D:heart rate)

Seven RCTs [4, 9, 40, 42–44, 52] reported the vital capacity of 712 participants (350 in the Qigong group and 362 in the control group), and the pooled result showed that the improvement of vital capacity in the Qigong groups was not significantly different from control groups (SMD = 0.07, 95% CI: -0.12 to 0.27, *P* = 0.46, I^2^ = 41%) (Fig. 4A). Subgroup analysis of both no intervention control and active control also showed no significant difference between the Qigong exercise and control groups (*P* = 0.55 and *P* = 0.47, respectively).

### Systolic blood pressure

Systolic blood pressure scores were analysed in three RCTs [4, 9, 44] that included a total of 428 participants (208 in the Qigong group and 220 in the control group). Meta-analysis showed that there was no significant difference between the Qigong exercise and control groups (MD =− 0.70 95% CI: − 3.50 to 2.09, *P* = 0.62, I^2^ = 26%) (Fig. 4B).

### Diastolic blood pressure

Diastolic blood pressure scores were analysed in three RCTs [4, 9, 44], including a total of 428 participants (208 in the Qigong group and 220 in the control group). The pooled results showed that compared with the control group, no significant difference was found in the Qigong group (MD=− 0.24, 95% CI: − 1.91 to 1.42, *P* = 0.77, I^2^ = 0%) (Fig. 4C).

### Heart rate

Three RCTs [4, 9, 44] analysed heart rate scores that included a total of 428 participants (208 patients in the Qigong group and 220 in the control group). The pooled result showed that there was no significant difference between the Qigong exercise and control groups (SMD = 0.00, 95% CI: − 0.19 to 0.19, *P* = 1.00, I^2^ = 0%) (Fig. 4D).

### Evaluation of psychological outcomes

#### Depression

**Fig. 5:**
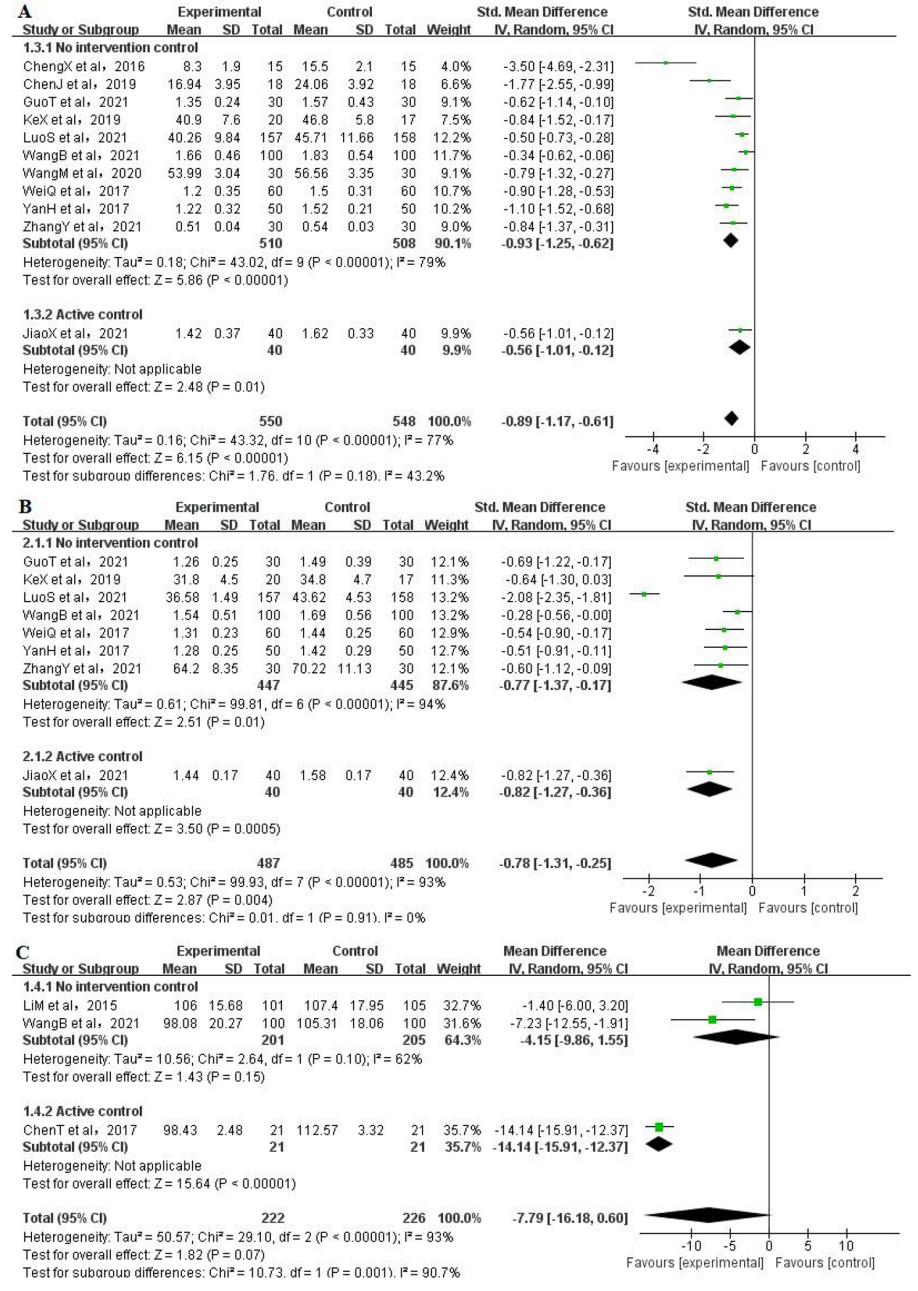
Meta-analysis of Qigong exercise on psychological outcomes (A:depression, B:anxiety, C:mood)

Eleven RCTs [39, 41–43, 45−46, 48−52] analysed the effect of Qigong interventions on depression, including a total of 1098 participants (550 in the Qigong group and 548 in the Control group). The pooled results showed that the difference was statistically significantly (SMD=− 0.89, 95% CI: − 1.17 to − 0.61, *P* < 0.00001). On the subgroup meta-analysis, both no intervention control (*P* < 0.00001) and active control subgroup (*P* = 0.01) showed significant differences. However, due to the still substantive heterogeneity among the studies (I^2^ = 79%) (Fig. 5A), a sensitivity analysis was conducted in which the included studies were excluded one by one. The heterogeneity was significantly reduced (I^2^ = 58%, *P* < 0.00001) when the study of ChengX et al., 2016, was removed (Supplementary Fig. 1). The funnel plot shows almost bilateral symmetry and is less likely to be influenced by publication bias (Fig. 5).

### Anxiety

Eight RCTs [39, 42–43, 48−52] analysed the anxiety of 972 participants (487 in the Qigong group and 485 in the Control group). The pooled meta-analysis showed that Qigong treated group had significantly lower anxiety than control group (SMD= − 0.78, 95% CI: − 1.31 to − 0.25, *P* = 0.004) with substantive heterogeneity (I^2^ = 93%) (Fig. 5B). Subgroup analysis also showed no significant effect in both the no intervention control (*P* = 0.01) and active control subgroup (*P* = 0.0005). But the heterogeneity in subgroup was still substantive (I^2^ = 94%). So sensitivity analysis was conducted and heterogeneity was significantly reduced when the study of LuoS et al., 2021 was removed (SMD= − 0.51, 95% CI: − 0.67 to − 0.35, *P* < 0.00001, I^2^ = 0%) (Supplementary Fig. 2).

### Mood

**Fig. 6:**
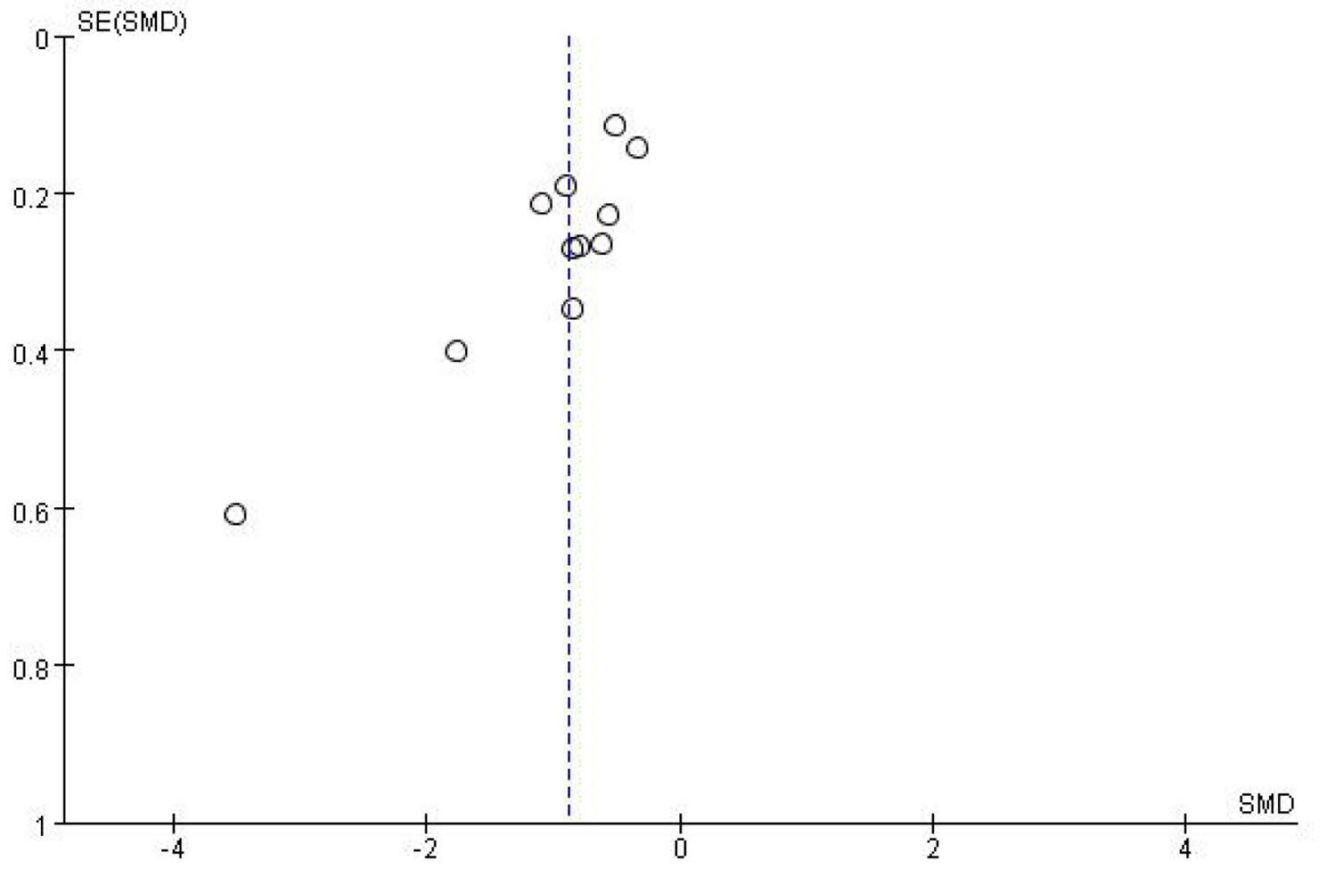
Funnel plot about meta-analysis of Qigong exercise on depression

Three RCTs [4, 47, 49] involving 448 participants (222 in the Qigong group and 226 in the Control group) analysed the effects of Qigong exercise on mood scores. The pooled results of the meta-analysis showed no significant changes between the Qigong exercise and control groups (MD=-7.79, 95% CI: -16.18 to 0.60, *P* = 0.07, I^2^ = 93%) (Fig. 6).

Subgroup analysis of no intervention control also showed no significant difference (*P* = 0.15, I^2^ = 62%), whereas the subgroup analysis of active control showed a significant improve in mood (*P* = 0.004).

### Sensitivity analyses

The results of sensitivity analysis were stable by model transformation between fixed effect model and random effects model and removing studies one by one.

## Discussion

The purpose of this systematic review and meta-analysis was to investigate whether Qigong could improve limb muscle strength, flexibility, cardiorespiratory endurance, vital capacity, blood pressure and heart rate and alleviate depression, anxiety and mood changes in college students when these parameters were evaluated with commonly used physical fitness and psychological assessments. The meta-analysis showed significant improvements in cardiorespiratory endurance (measured by the step test) and flexibility (measured by the sit-and-reach test) with Qigong exercise. In addition, there was a significant reduction in depression and anxiety symptoms. However, there was no significant effect of Qigong exercise on vital capacity, blood pressure, heart rate, muscle strength (measured by the standing long jump test and hand grip force test) or mood.

The significant improvement in flexibility was also supported by Wehner C et al. [29]. Based on the included studies, twice a day, five days a week, for three months, Badujin and Yijinjing are recommended for improving trunk flexibility [42–43]. However, due to unexplained high heterogeneity of uncertain causes, we need to be cautious of this result.

We used step test to measure cardiorespiratory endurance. The meta-analysis showed that Qigong was beneficial for cardiorespiratory endurance. The included studies suggested that Yijinjing twice a day, more than 5 days a week for 3 months and Wuqinxi 5 days a week for 4 months are beneficial to improve the cardiopulmonary endurance of college students [42, 52]. However, given the unexplained high heterogeneity, we need to be cautious of these results.

Depression and anxiety have been widely studied in previous epidemiological studies looking at the relationship between exercise and mental health [53–54]. The results of this review are consistent with the conclusions of a previously published review suggesting that Tai Chi and Qigong exercises contribute to reducing anxiety and depression symptoms [26, 55–56]. Specifically, effect size estimates with respect to the effects of Qigong ranged from − 0.52 to − 1.94 for depression levels and from − 0.39 to − 0.78 for anxiety levels. The effective mechanism of Qigong may be attributed to Qigong training increasing excitation of the middle cerebral cortex, which has been shown to modulate the hypothalamic-pituitary-adrenal axis [57–58], monoamine neurotransmitter [57], brain-derived neurotrophic factor [57] and adiponectin [59] to alleviate severe psychological symptoms, such as anxiety and depression.

No significant changes in other physical outcomes, including limb strength, vital capacity, blood pressure and heart rate, were observed. One possible explanation for this result is that all participants enrolled in this trial were young (aged 18–25 years old) and apparently healthy college students. Ainsworth et al. [60] classified the intensity of Qigong, such as Tai Chi, as moderate intensity, equivalent to 4 metabolic equivalents, so Qigong may only be appropriate for people with chronic diseases and not for healthy people with relatively high baseline health levels. In addition, it is possible that the 12-week intervention period for Qigong exercise is not sufficient to identify significant differences in muscular strength, vital capacity, blood pressure or heart rate for an apparently healthy college student population.

The current meta-analysis mainly has the following shortcomings. First, considering that many Qigong studies originated in China and were published in Chinese, the research languages included were limited to English and Chinese, but this still inevitably leads to some omissions. Therefore, future research should include a wider range of learned languages. Second, in this meta-analysis, most of the included trials had flaws in methodological design, mainly including the method of randomization, allocation concealment, and insufficient reporting of blinding. Third, most of the included studies did not provide data on whether participants continued to practice Qigong after the intervention period. Due to a lack of follow-up data, the long-term effect of Qigong on the physical and mental health of college students is still unclear.

## Conclusion

This systematic review and meta-analysis suggests that Qigong exercise is advantageous for college students in terms of improving physical fitness (flexibility and cardiorespiratory endurance) and alleviating depression and anxiety. However, given the low methodological quality, accurate conclusions cannot be drawn thus far, and the findings need to be interpreted with caution. More large, well-designed randomized controlled trials are needed in the future.

## Electronic supplementary material

Below is the link to the electronic supplementary material.

Supplementary Material 1: **Figure S1**. Sensitivity analysis of the effect of Qigong interventions on depression

Supplementary Material 2: **Figure S2.** Sensitivity analysis of the effect of Qigong interventions on anxiety

Supplementary Material 3: PRISMA checklist

## Data Availability

All data are included in this manuscript and its supplementary files.

## Abbreviations

RCTs: Randomized controlled trials
MD: Mean difference
SMD: Standardized mean differences
SD: Standard deviation
CENTRAL: Cochrane Central Register of Controlled Trials
CNKl: China National Knowledge Information Database
PRISMA: Preferred Reporting Items for Systematic Reviews and Meta-Analyses
SDS: Self-rating depression scale
CES-D: Center for epidemiologic studies depression scale
HAMD: Hamilton depression scale
SAS: Self-rating anxiety scale
PMOS: Profile of mood states.
