# Supplementary figures and images for: Effects of qigong exercise on the physical and mental health of college students: a systematic review and Meta-analysis
Source: BMC Complement Med Ther. 2022 Nov 8;22:287. doi: 10.1186/s12906-022-03760-5 (PMC9641907; doi:10.1186/s12906-022-03760-5)

| 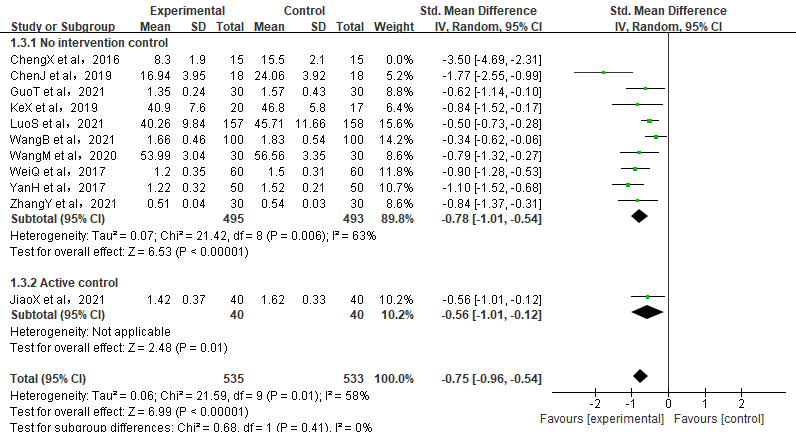 |
| --- |
| **Figure S1.** Sensitivity analysis of the effect of Qigong interventions on depression |

Supplement: Supplementary file 2 — Supplementary Material 2: Figure S2. Sensitivity analysis of the effect of Qigong interventions on anxiety [file 12906_2022_3760_MOESM2_ESM.docx]

| 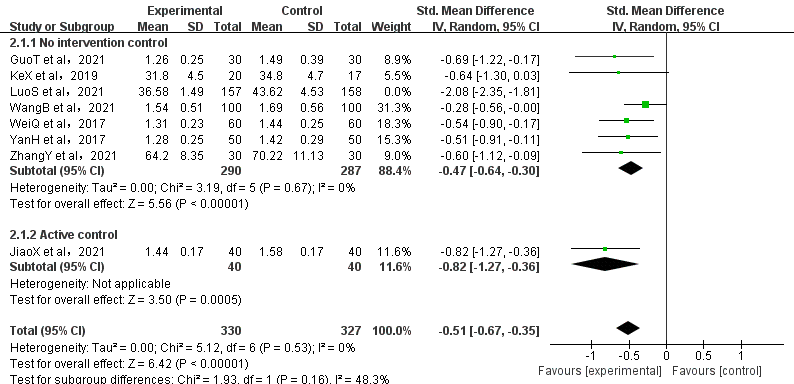 |
| --- |
| **Figure S2.** Sensitivity analysis of the effect of Qigong interventions on anxiety |

Supplement: Supplementary file 3 — Supplementary Material 3: PRISMA checklist [file 12906_2022_3760_MOESM3_ESM.docx]
